# Endotype-driven treatment in chronic upper airway diseases

**DOI:** 10.1186/s13601-017-0157-8

**Published:** 2017-07-12

**Authors:** Glynnis De Greve, Peter W. Hellings, Wytske J. Fokkens, Benoit Pugin, Brecht Steelant, Sven F. Seys

**Affiliations:** 1 Department of Otorhinolaryngology-Head and Neck Surgery, UZ Leuven, Louvain, Belgium; 2 Department of Otorhinolaryngology, Academic Medical Center, Amsterdam, The Netherlands; 3 Upper Airways Research Laboratory, Department of Otorhinolaryngology-Head and Neck Surgery, Ghent University, Ghent, Belgium; 4 Laboratory of Clinical Immunology, Department of Immunology and Microbiology, KU Leuven, Herestraat 49/PB811, 3000 Louvain, Belgium

**Keywords:** Rhinitis, Chronic rhinosinusitis, Phenotype, Biomarker, Biologicals, Precision medicine, Personalised medicine

## Abstract

Rhinitis and rhinosinusitis are the two major clinical entities of chronic upper airway disease. Chronic rhinosinusitis (CRS) and allergic rhinitis (AR) affect respectively up to 10 and 30% of the total population, hence being associated with an important socio-economic burden. Different phenotypes of rhinitis and CRS have been described based on symptom severity and duration, atopy status, level of control, comorbidities and presence or absence of nasal polyps in CRS. The underlying pathophysiological mechanisms are diverse, with different, and sometimes overlapping, endotypes being recognized. Type 2 inflammation is well characterized in both AR and CRS with nasal polyps (CRSwNP), whereas type 1 inflammation is found in infectious rhinitis and CRS without nasal polyps (CRSsNP). The neurogenic endotype has been demonstrated in some forms of non-allergic rhinitis. Epithelial barrier dysfunction is shown in AR and CRSwNP. Emerging therapies are targeting one specific pathophysiological pathway or endotype. This endotype-driven treatment approach requires careful selection of the patient population who might benefit from a specific treatment. Personalized medicine is addressing the issue of providing targeted treatment for the right patient and should be seen as one aspect of the promising trend towards precision medicine. This review provides a comprehensive overview of the current state of endotypes, biomarkers and targeted treatments in chronic inflammatory conditions of the nose and paranasal sinuses.

## Background

Persistent rhinitis and chronic rhinosinusitis (CRS) are the two major clinical entities of chronic upper airway disease. Worldwide questionnaire-based surveys show that allergic rhinitis affects up to 30% of the global population, whereas CRS is present in over 10% of the European population [1, 2]. Upper airway diseases are often associated with comorbidities such as asthma or COPD [3, 4]. The upper and lower airways cannot be separated from each other and immune modulating drugs, such as allergen immunotherapy and biologicals, affect both airway compartments.

Uncontrolled disease has been reported in 35–40% of patients with chronic upper airway disease and has a substantial impact on the patient’s social, physical and economic health. The reasons for an uncontrolled disease are related to disorder-, diagnosis-, treatment- or patient-associated factors. The relative importance of these factors is unclear, specialists agree that optimal disease management approaches are needed [5–7].

Precision medicine is proposed to address this global issue by providing customized and individualized care based on the unique immunologic, genetic and psychosocial profile of the patient [8]. The concept of precision medicine is based on four pillars: *personalized care* with tailored diagnostic and therapeutic approaches, *prediction* of disease progression and success of treatment, *prevention* of disease and *participation* of the patient to achieve good adherence and optimal efficacy of the given treatment.

To fully implement precision medicine into daily practice, disease management based on disease control and phenotyping needs to be complemented with disease endotyping. For decades, to determine the best-fit treatment, a phenotype is being assigned to the patient based on clinical symptoms, atopy status and the presence of nasal polyps (for CRS patients). This approach is generally carried out almost entirely regardless of the underlying pathophysiological mechanisms. In complex diseases with mixed pathophysiologies, a phenotype-driven treatment is not always sufficient to obtain optimal control. Endotype classification based on thorough investigation of the underlying pathophysiological mechanisms is therefore gaining more interest. Endotyping will provide more insight in the inter-individual variability of clinical presentation and treatment response in patients with identical phenotypes. In addition, endotyping might in the future guide the decision making process of targeted treatments [9]. In order to make endotype-driven treatment a clinical applicable approach in daily practice, identification of measurable biological indicators, or so called “biomarkers”, is needed [10]. The ideal biomarker serves as a signature of a well-defined endotype and is easily measurable, reproducible and affordable [11].

Currently we are in the era of extensive research towards identification of biomarkers and endotype-driven treatments. Research on endotyping is also performed for asthma and cancer and is well ahead of endotyping in upper airway diseases. The aim of the current review is to provide a comprehensive overview of the current state of endotypes, biomarkers and biological treatment in rhinitis and CRS. Since biomarkers can be used for many applications, only those that are (potentially) of valuable for the diagnosis or prediction of treatment response will be reviewed. Subsequently, current or potential treatment strategies targeting specific endotypes will be discussed.

## Endotypes and biomarkers in upper airway diseases

Rhinitis is characterized by inflammation of the nasal mucosa causing nasal obstruction, rhinorrhoea, sneezing and pruritus [12]. Three main phenotypes of rhinitis are described: allergic rhinitis (AR), infectious rhinitis and non-allergic non-infectious rhinitis (NAR). The latter phenotype can be subdivided in many subphenotypes such as idiopathic rhinitis (IR), hormonal rhinitis, gustatory rhinitis, drug-induced rhinitis, rhinitis of the elderly, atrophic rhinitis and occupational rhinitis [13]. In CRS the mucosal inflammation affects the nose and paranasal sinuses and is characterized by nasal obstruction and discharge, loss of smell and/or facial pain, which lasts longer than 12 weeks [14]. Traditionally a phenotype is addressed to the patient according to the presence (CRSwNP) or absence (CRSsNP) of nasal polyps on nasal endoscopy or radiological imaging.

**Fig. 1:**
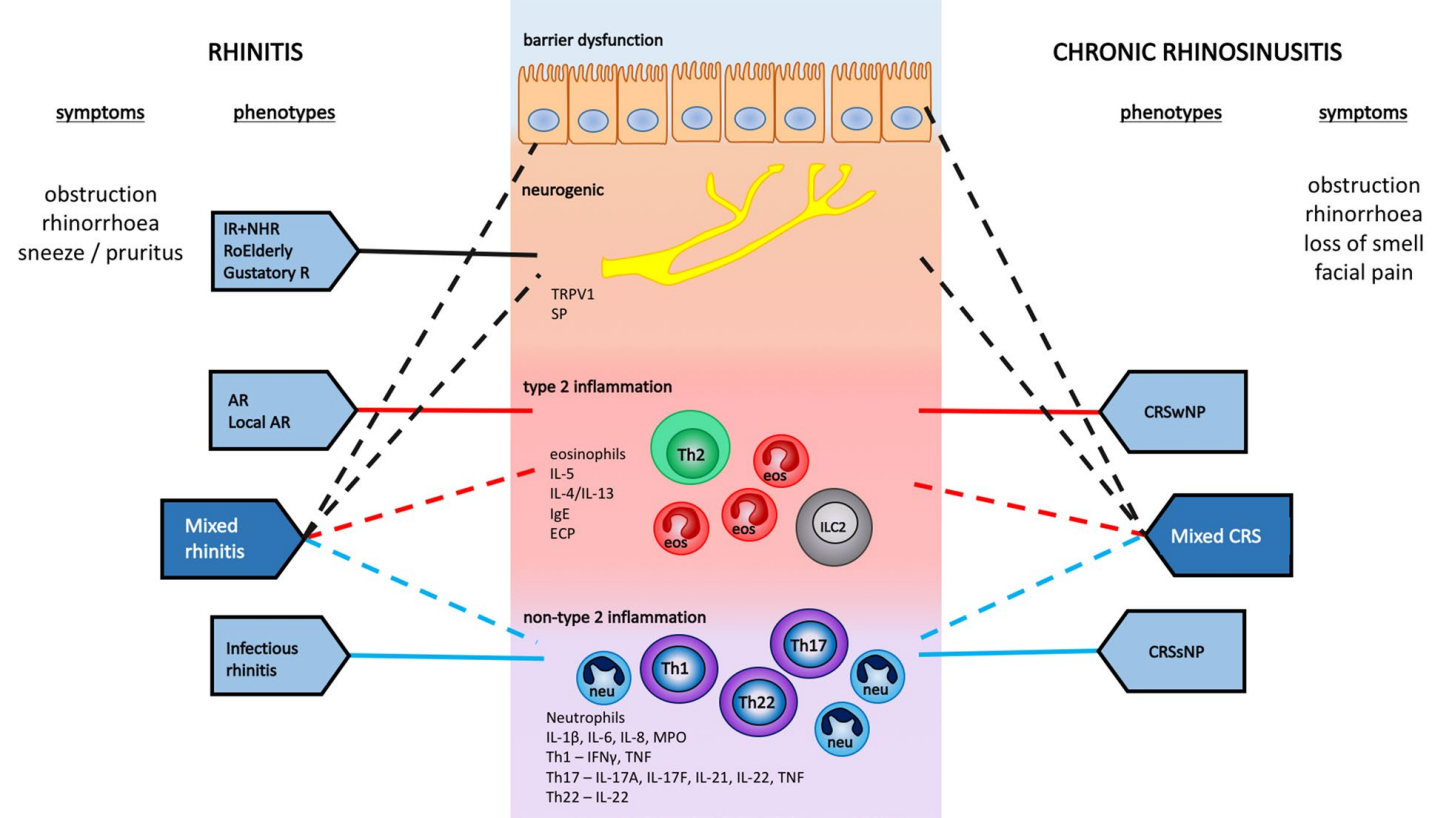
Overview of endotypes and phenotypes in rhinitis and chronic rhinosinusitis. Endotype predominantly underlying the phenotype, *solid lines*; endotype potentially contributing to the phenotype, *dashed lines*. *AR* allergic rhinitis; *CRSsNP* chronic rhinosinusitis without nasal polyps; *CRSwNP* chronic rhinosinusitis with nasal polyps; *IR* idiopathic rhinitis; *RoElderly* rhinitis of the elderly; *Gustatory R* gustatory rhinitis

A specific phenotype can be indicative for the presence of one particular endotype. However, one or mixed endotype(s) can also underlie different phenotypes in upper airway diseases, hence making clear distinction of endotypes more complex. Since the underlying pathophysiological events of both rhinitis and CRS are located at the upper airway mucosal lining, they share common endotypes (Fig. 1).

### Type 2 inflammation

**Fig. 2:**
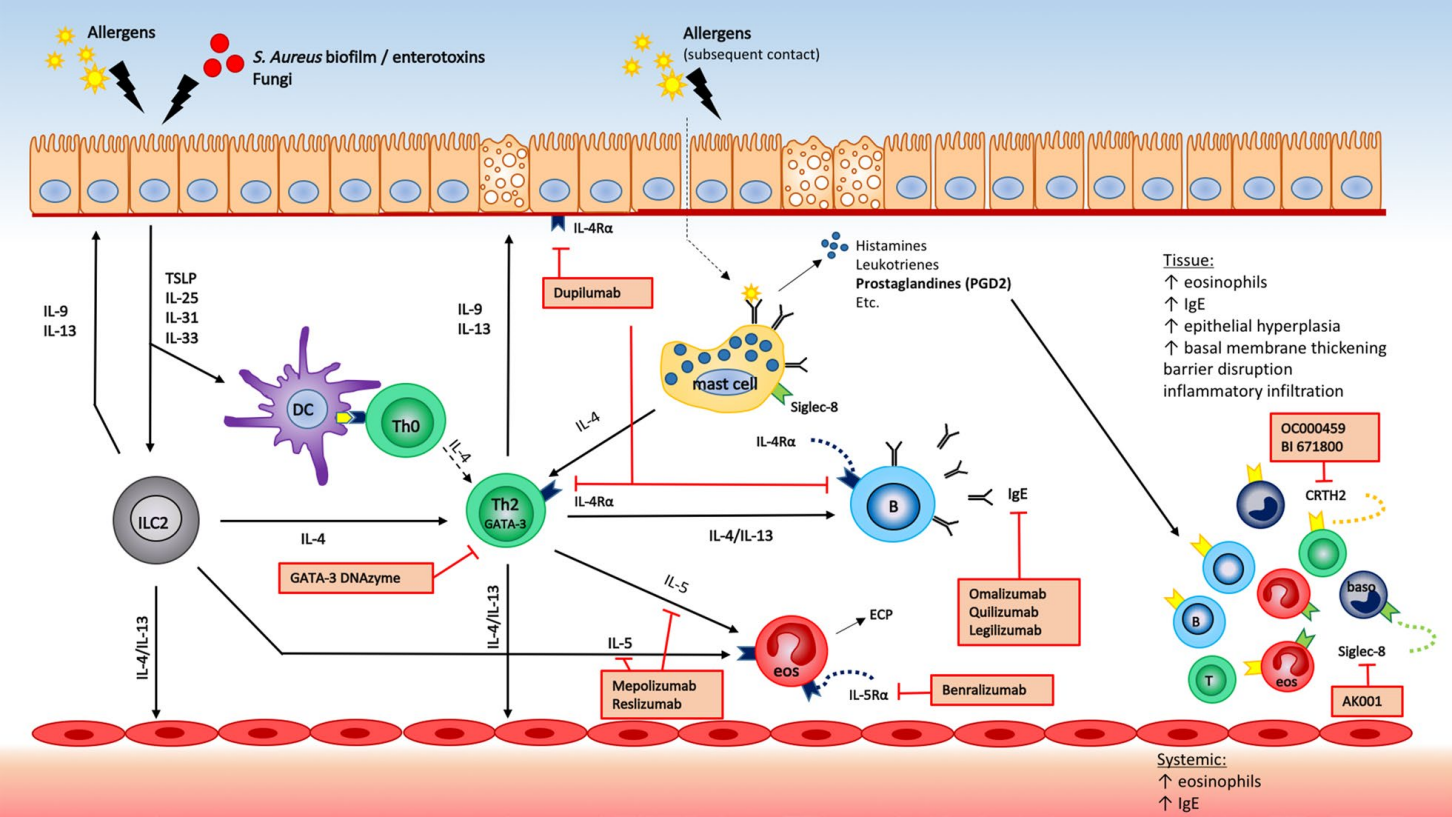
Type 2 inflammation and biologicals. *B* B cell; *baso* basophil; *DC* dendritic cell; *ECP* eosinophilic cationic protein; *eos* eosinophils; *ILC2* type 2 innate lymphoid cell; *Th* T helper cell

Type 2 inflammation is characterized by the presence of eosinophils and type 2 cytokines IL-4, IL-5, IL-9 and IL-13, derived from Th2 cells and type 2 innate lymphoid cells (ILC2), in peripheral blood or nasal mucosa [15]. IL-25, IL-31, IL-33 and thymic stromal lymphopoietin (TSLP) secreted by epithelial cells are known to induce or enhance type 2 driven inflammation [16]. In sensitized individuals, contact with allergens activates mast cells via immunoglobulin E (IgE) dependent mechanisms [9] (Fig. 2).

Type 2 inflammation is a major feature of AR, local allergic rhinitis (LAR) and CRSwNP, hence the most common and studied endotype in upper airway diseases (Table 1). Topical and/or oral corticosteroids are the first-line treatment for these patients and are shown to reduce eosinophils in nasal mucosa [17–19]. However, treatment with corticosteroid can be insufficient to fully control the inflammation.

**Table 1:** Potential diagnostic biomarkers

Endotypes	Allergic rhinitis	Idiopathic rhinitis	Infectious rhinitis	CRSwNP	CRSsNP
***Type 2 inflammation***					
Serum	Eosinophils* Total IgE Specific IgE ECP*			Eosinophils* Total IgE* IgE/SE-IgE* ECP*	
Nasal fluids	Total/specific IgE* IL-5* IL-4, IL-13* Eosinophils*			Total/specific IgE* IL-5* IL4, IL-13 ECP/MPO ratio* ECP*	
***Non-*** ***type 2 inflammation***					
Nasal lavage/fluids			IL-1P, IL-6, IL-8, MPO IFNγ TNFα		IL-1P, IL-6, IL-8, MPO* IFNγ* IL-17, IL-22, TNFα*
Nasal biopsy			Neutrophils		Neutrophils
***Neurogenic endotype***					
Nasal fluids		SP			
Nasal biopsy		TRPV1			
***Barrier dysfunction***					
Nasal biopsy	↓TER		↓TER	↓TER	
	↓TJ		↓TJ	↓TJ	↓TJ

*ECP* eosinophilic cationic protein; *MPO* myeloperoxidase; *TER* transepithelial resistance; *TJ* tight junctions; *TRPV1* transient receptor potential cation channel subfamily V receptor 1

* Also detectable in nasal biopsies

AR is predominantly defined by the type 2 endotype. Allergen exposure through nasal mucosa triggers the type 2 inflammatory cascade leading to a Th2-dominant milieu with eosinophilia and specific IgE production [20]. The diagnosis of AR is based on clinical features and allergen sensitization. A positive skin prick test (SPT) or ImmunoCap test, using rather arbitrary cut-off values of wheal diameter ≥3 mm and serum IgE ≥ 0.35 KU/l, are used to confirm atopic sensitization [21]. A negative SPT however, does not exclude presence of type 2 inflammation, e.g. LAR. Additional nasal allergen provocation test and nasal secretion sampling for specific IgE detection can provide evidence of type 2 inflammation [22]. In addition, serum total IgE, eosinophilic cationic protein (ECP, activation marker of eosinophils) and eosinophils have been proposed as diagnostic biomarkers with corresponding cut-off values of 98.7 IU/mL, 24.7 µg/mL and 4.0%, respectively, with sensitivities ranging from 55.7 to 75.2% and specificities from 69.7 to 74.4% [23]. Type 2 cytokines IL-4, IL-5 and IL-13 are also detectable in nasal fluids [24]. So far, no validated diagnostic cut-off values are available.

Unlike AR, type 2 inflammation in CRSwNP is characterised by polyclonal IgE formation and is usually not linked to atopy [25]. This endotype is the most common one in white people from Europe and US with eosinophilic CRSwNP [26–28].

In research setting, the diagnosis of eosinophilic CRSwNP is either based directly on tissue eosinophilia (>5 eosinophils/high power field or indirectly on ECP/myeloperoxidase (MPO) ratio (>1) determined on nasal biopsies [26, 27, 29]. A cut-off value of >10 eosinophil/HPF however is clinically more relevant to assess its impact on quality of life (QoL) [30]. One cross-sectional study with 51 patients shows that serum eosinophilia values of >0.3 × 10^9^ L^−1^ or 4.4% of white blood cells have a positive predictive value and negative predictive value of 79 and 67%, respectively [31]. Furthermore, type 2 cytokines IL-4, IL-5 and IL-13 are detectable in nasal secretions. Surprisingly IL-13 levels were elevated in samples of healthy controls compared to those of patients with CRSsNP and CRSwNP, and IL-4 levels showed no significant raise. IL-5 was significantly higher in presence of nasal polyps compared to CRSsNP and healthy controls and might therefore be a useful biomarker to predict ongoing type 2 inflammation in CRSsNP patients [32]. Hence, all the above-mentioned markers, corresponding cut-off values and predictive values need to be validated in larger cohorts of patients.

Some studies evaluated the potential of type 2 inflammatory markers as prognostic biomarkers. Higher levels of mucosal and/or blood eosinophilia and presence of comorbid asthma are correlated with poor outcome in terms of QoL, recurrence of NP after sinus surgery and disease severity [29, 30, 33–35]. Other type 2 inflammation markers such as IgE, ECP and IL-5 are also predictive for recurrence of CRSwNP [28, 36].

*Staphylococcus aureus* is found in around 60% of CRS patients with eosinophilic inflammation and NP [37]. Whether *S. aureus* is an initiator or amplifier in CRS is a matter of debate. *S. aureus* biofilms are documented in CRS patients with more severe disease and worse post-operative outcome [38, 39]. Biofilms make bacteria more resistant to therapy with antibiotics, thus allowing them to penetrate submucosally and initiate type 2 inflammation. IL-4 and IL-13 may also compromise the immune response to *S. aureus* through the suppression of human β-defensin released in skin and mucosa [40, 41]. Importantly, *S. aureus* produces enterotoxins (SE) which can act as superantigens. These superantigens have the unique ability to amplify the type 2 inflammation through interaction with T-cells via the T cell receptors, as a result of their unrestricted antigen specificity, and in turn leading to the production of polyclonal IgE against SE (SE-IgE) [42]. Presence of SE-IgE itself is a risk factor for the development of comorbid asthma [26, 36, 37, 43, 44], and NP recurrence after surgery [28].

### Non-type 2 inflammation

**Fig. 3:**
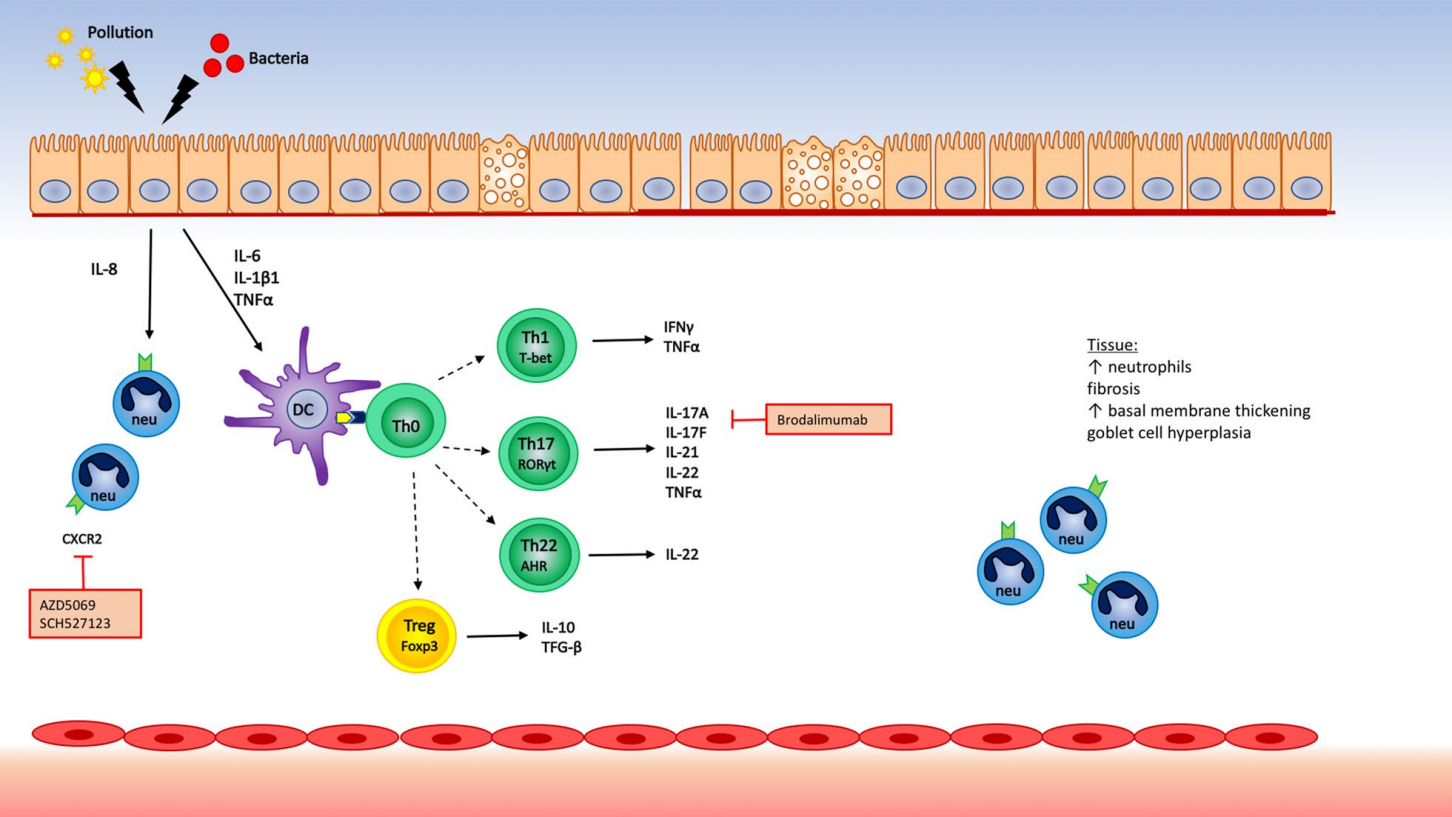
Non-type 2 inflammation and biological. None-type 2 hosts different T helper subsets. Th1 cells, Th17 and Th22 cells characterized by their individual transcription factors (T-bet, RORyt, AHR) are responsible for Th1, Th17 and Th22 cytokines respectively. Regulatory T cells suppress the immune response via production of IL-10 and TGF-β. *DC* dendritic cell; *neu* neutrophils; *Th* T helper cell; *Treg* T regulatory cell

Non-type 2 inflammation is mainly characterized by neutrophils in nasal mucosa [27, 44, 45]. Neutrophilic inflammation can be triggered by infections or chronic irritation, such as air pollution. This leads to dysregulation of the innate immune system and activation of the IL-17 pathway with recruitment of neutrophils to the nasal mucosa, which is known to be mediated via IL-8 [46–48]. In addition, type 1 immune response, metabolic and epigenetic factors, or the activation of the epithelial-mesenchymal trophic unit may lead to extensive remodeling without any inflammation, have been identified as modulating factors of the neutrophilic inflammation [49, 50] (Fig. 3).

Research on endotyping in non-type 2 inflammatory diseases lags well behind the type 2 inflammatory diseases, and so far no endotype-driven treatment has been proven to be effective. Since tissue neutrophilia is associated with reduced clinical response to corticosteroids, further exploration of none-type 2 is needed [18, 51].

Infectious rhinitis is associated with neutrophilic inflammation with increased pro-inflammatory cytokines IL-1β, IL-6, IL-8, interferon (IFN) γ, tumor necrosis factor (TNF)α and MPO [52, 53]. These cytokines can be detected in nasal lavage samples during acute upper respiratory tract infection.

CRSsNP is generally associated with neutrophilic inflammation and increased levels of IFNγ and IL-17, although it has also been documented in CRSwNP, especially in the Asian population [26]. Based on the study of Tomassen et al. three non-type 2 subendotypes were identified in patients with CRS: (1) neutrophilic inflammation characterized by pro-inflammatory cytokines IL-1β, IL-6, IL-8 and MPO; (2) Th17- or Th22- driven inflammation characterized by IL-17, IL-22 and TNFα; (3) Th1-driven inflammation characterized by IFNγ [44]. A combination of these subendotypes are often documented in both CRSsNP and CRSwNP, hence resulting in a mixed endotype [26]. During early stage CRSsNP, increased levels of transforming growth factor (TGF) β1 in sinus tissue compared to turbinate tissue from controls were reported, suggesting that TGFβ1 plays a pivotal role in initiating collagen production and remodelling process [54]. In CRSwNP patients with non-recurrent disease, higher levels of IFNγ indicative of Th1-driven inflammation were found compared to those with recurrent disease [28].

### Neurogenic activation

**Fig. 4:**
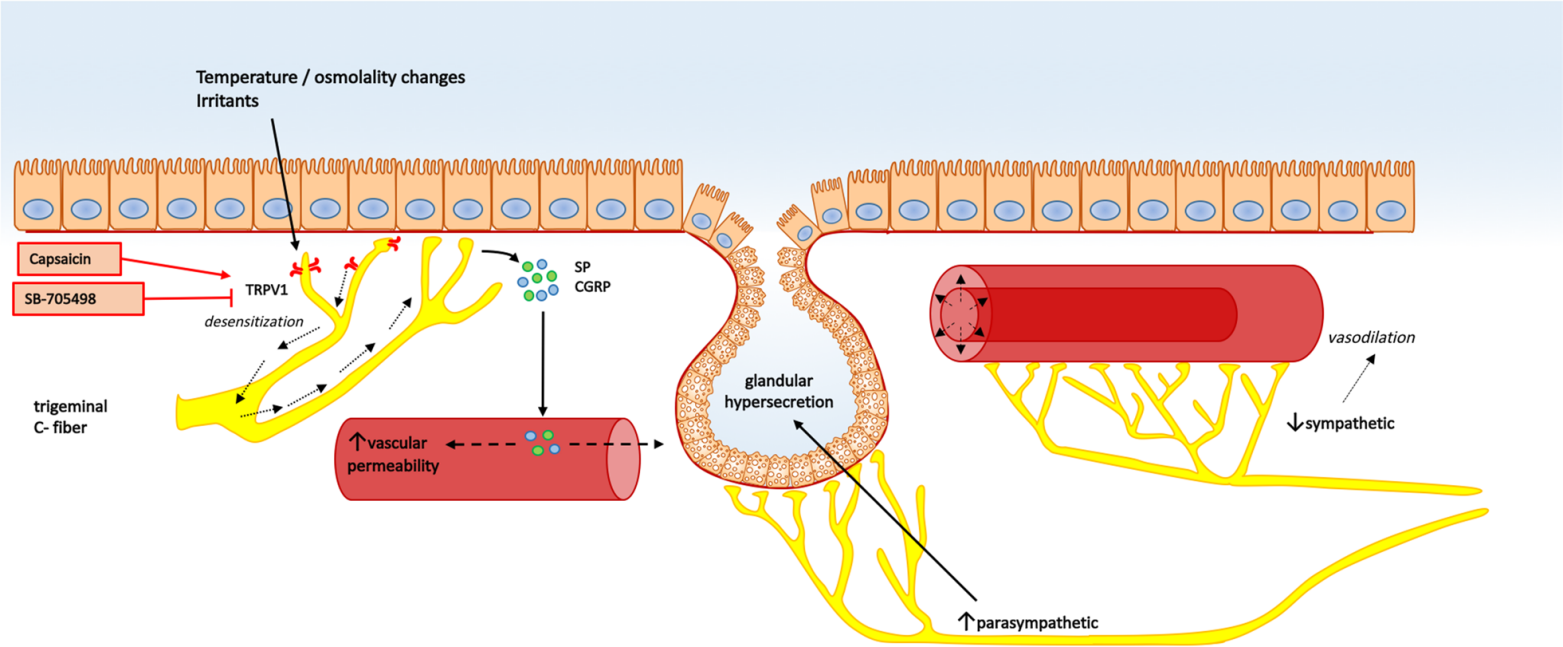
Neurogenic endotype and biologicals. TRPV1 overexpression resulting in nasal hyperreactivity on temperature and/or osmolality changes and irritants (*left side*). Dysautonomia (*right side*). *CGRP* calcitonin G-related peptide; *SP* substance P; *TRPV1* transient receptor potential vanniloid 1

Dysfunction of the neuronal system of the nose is underlying different subphenotypes of NAR such as idiopathic rhinitis (IR), gustatory rhinitis and rhinitis of the elderly [55]. Two pathophysiological mechanisms are proposed: 1/overexpression of transient receptor potential (TRP) channels and associated nasal hyperreactivity (NHR) and 2/imbalance of sympathetic and parasympathetic system [56] (Fig. 4).

NHR is present in two-thirds of patients with IR, i.e. aberrant reactivity of the nasal mucosa to common environmental stimuli such as smoke, chemical pollutants, strong odors and temperature and humidity alterations resulting in defensive responses such as sneezing, rhinorrhoea and nasal congestion [57]. The nasal mucosa is equipped with C-fibers, a specific type of sensory nerve, which express TRP channels on their endings. These nerves can be activated by non-allergic triggers, such as environmental irritants, alterations in temperature or osmolality, and can subsequently induce the release of neuropeptides like substance P (SP) and calcitonin G-related peptide (CGRP). These neuropeptides induce increased vascular permeability and glandular hypersecretion resulting in the above-mentioned rhinitis symptoms [56].

Van Gerven et al. demonstrated an association between IR and TRP subfamily V receptor 1 (TRPV1) overexpression in nasal mucosa. In addition, increased SP levels were found in nasal secretions of IR patients, supporting a causative role of the nociceptive TRPV1-SP signaling pathway [57]. Evaluation of SP in nasal secretions could potentially serve as a diagnostic biomarker for IR. However, more easy and rapid tests are required to allow identification of IR patients in daily practice. The cold-dry air provocation test (CDA) is a diagnostic test for NHR with a high sensitivity and specificity, thus providing a reliable, easy, well-tolerated but most importantly non-invasive test using natural stimuli compared to a more labor intensive nasal sampling [58].

Moreover, the imbalance of the autonomous nervous system, also called “dysautonomia” might also contribute to the pathophysiological mechanism of NAR. Parasympathetic and sympathetic activity results in vasodilation and vasoconstriction, respectively. An imbalance of these components with loss of sympathetic tone and relative increased parasympathetic activity, results in vasodilation, increased mucosal blood flow and glandular hypersecretion [56]. Although the pathophysiology of rhinitis of the elderly is not clear, dysautonomia is thought to be the causative mechanisms leading to the typical clear rhinorrhea [13]. So far no biomarkers are evaluated to identify patients with dysautonomia.

### Epithelial barrier dysfunction

**Fig. 5:**
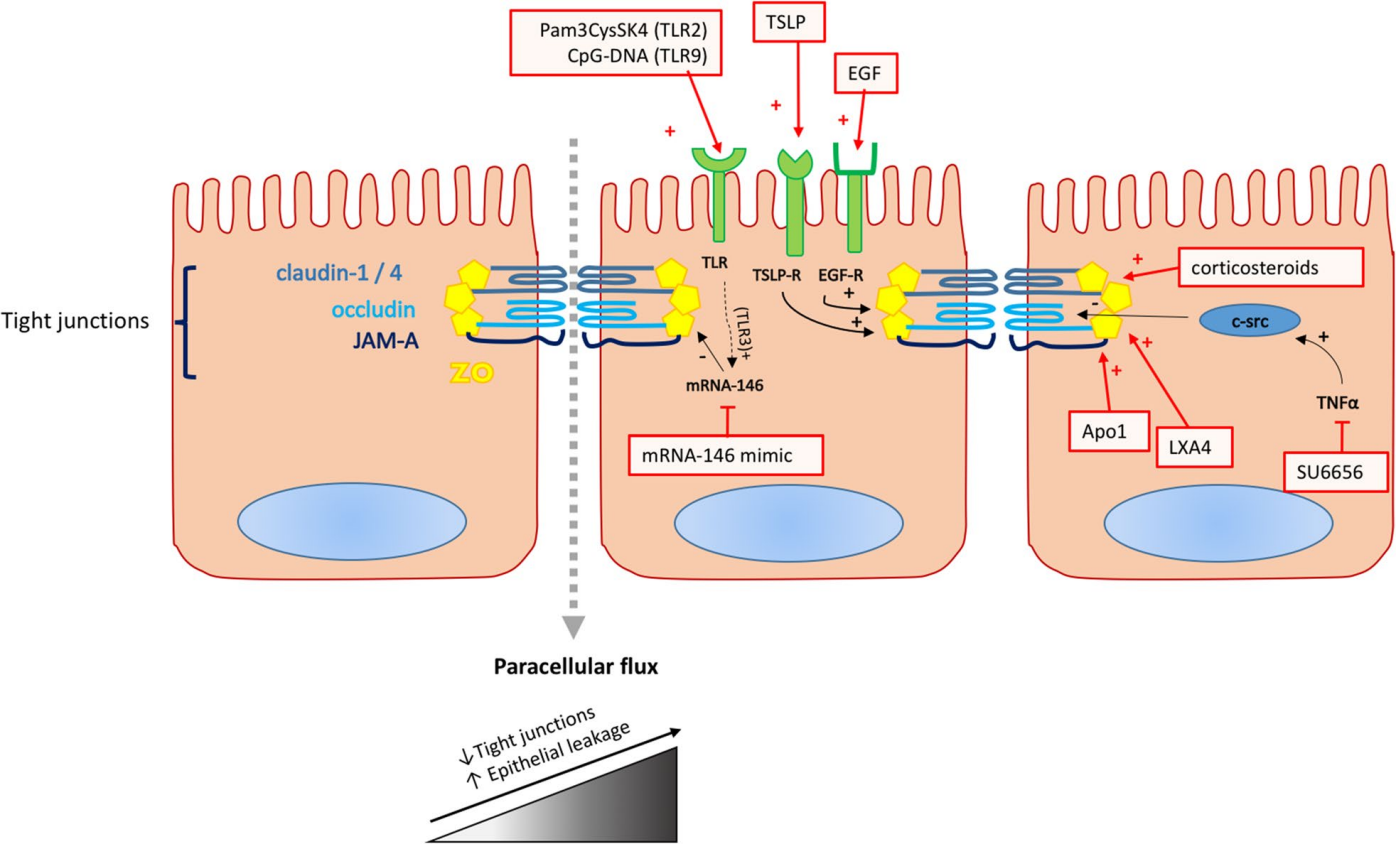
Barrier dysfunction and potential biomarkers. *EGF* epidermal growth factor; *EGF*-*R* epidermal growth factor receptor; *JAM*-*A* junctional adhesion molecule A; *TLR* toll-like receptor; *ZO* zona occludens

The epithelial lining forms the first barrier for exogenous pathogens or harmful particles. Besides being a physical barrier and maintaining mucociliary clearance, it modulates the innate immune response by through cytokine and chemokine production [59]. Proper functioning of the physical barrier is supported by dynamic junctional complexes that connect epithelial cells to one another and regulate paracellular flux of molecules of a certain size. Tight junctions are apically located epithelial junctions, consisting of different transmembrane proteins such as claudin-1, claudin-4, occludin and junctional adhesion molecule A, which are connected to intracellular proteins like zonula occludens-1 (ZO-1) among others (Fig. 5).

A defective epithelial barrier has been documented in various chronic airway diseases, such as AR, CRS and asthma, and is associated with chronicity and severity of the inflammation [60–62]. A leaky epithelium was documented in CRSwNP due to decreased expression of occludin and claudin-4 on nasal biopsies [61]. Similarly a disrupted tight junction arrangement in AR was found to be due to decreased expression of occludin and ZO-1 [62].

Whether epithelial barrier dysfunction is a primary genetic event or a secondary phenomenon resulting from inflammation is not clear. A dysfunctional epithelial barrier results in increased permeability for foreign particles allowing them to migrate to the submucosal region, hence making it more vulnerable for inflammation. In addition, there is evidence that IL-4 disrupts epithelial integrity suggesting that type 2 inflammation can contribute to epithelial dysfunction [62]. Since epithelial barrier dysfunction is part of AR and CRS, restoring the barrier integrity may become a useful treatment approach. So far, no easy methods are available to evaluate barrier function in patients with upper airway disease.

## Treatment of chronic upper airway disease

### Therapies targeting type 2 inflammation

#### Targeting IgE-pathway

Omalizumab, a recombinant humanized anti-IgE monoclonal antibody (mAb), binds circulating IgE on its high-affinity receptor (FcεRI) preventing it to become cell-bound on effector cells such as mast cells, basophils, dendritic cells and eosinophils. Subsequently the expression of FcεRI reduces on the effector cells [63–65]. Omalizumab is approved by the European and US regulatory authorities for the treatment of severe allergic asthma and is currently under investigation for its use in the treatment of allergic rhinitis and CRS (Table 2).

**Table 2:** Targets and potential treatments according to the specific endotypes and phenotypes

Endotypes-targets	Allergic rhinitis	Idiopathic rhinitis	Infectious rhinitis	CRSwNP	CRSsNP
***Type 2 inflammation***					
IgE	Omalizumab (sc/iv)			Omalizumab (sc)	
	Legelizumab			Legelizumab	
Ml prime mIgE	Quilizumab			Quilizumab	
IL-5	Mepolizumab			Mepolizumab (iv)	
	Reslizumab			Reslizumab (sc)	
IL-5Ra	Benralizumab			Benralizumab	
IL-4/13	Dupilumab			Dupilumab (sc)	
CRTH2	OC000459 (po)			OC000459	
	BI 671800 (po)			BI671800	
GATA-3	GATA-3 spec.DNAzyme			GATA-3 spec.DNAzyme	
Siglec-8	AK001				
***Non-type 2 inflammation***					
Neutrophilic inflammation					
Th1					
Th17/Th22			Brodalumab		Brodalumab
***Neurogenic endotype***					
TRPV1		Capsaicin (in) SB-705498 (IN)			
***Barrier dysfunction***					
None					

*IN* intranasal; *IV* intravenous; *po* per os; *sc* subcutaneous

Cumulative evidence exists that treatment with omalizumab is safe, well-tolerated and effective in reducing symptoms and rescue medication use in AR [66–73]. Adding omalizumab to allergen immunotherapy (AIT) for the treatment of allergic rhinitis with or without co-morbid allergic asthma appears to be superior to either treatments alone [72, 74, 75]. Combination therapy shows superiority in the treatment of polysensitized patients, due to its allergen-independent therapeutic effect [76]. Furthermore, it has a protective effect on the development of adverse events of AIT, hence allowing rush-immunotherapy treatment with higher dose regimens and a shorter treatment course [72, 76, 77]. In patients with persistent AR and concomitant asthma, omalizumab is effective in preventing asthma exacerbation and improving quality of life [78, 79]. Unfortunately, omalizumab has no long-term effect [80, 81] unlike AIT, which remains the sole curative approach nowadays.

In CRSwNP patients and co-morbid asthma omalizumab showed reduced upper and lower airway symptoms, endoscopic nasal polyp score as well as less needs for further medical or surgical treatments [25, 82, 83]. On the other hand, one trial revealed that the molecule had a small and clinically irrelevant effect on CRS [84]. This trial however was underpowered and the presence of NPs was not taken into account. This emphasizes the importance of endotyping to properly select patients who will benefit most from anti-IgE treatment.

New promising biologicals targeting IgE are being developed with the aim of improving anti-IgE treatment. One such is ligelizumab, an anti-IgE mAb with greater affinity for IgE compared to omalizumab [85]. A second one is quilizumab, a mAb targeting the M1 epitope on membrane IgE [86]. Studies are currently running to assess their safety and efficacy in the treatment of asthma.

#### Targeting IL5-pathway

IL-5 is a key mediator in type 2 eosinophilic inflammation [26, 28, 36, 87]. It is responsible for survival, maturation and activation of eosinophils at the bone marrow and the site of inflammation [87–89]. To interfere with the IL-5 pathway, novel biologicals are developed targeting IL-5 or its receptor IL-5Rα on the effector cells. Mepolizumab and reslizumab are both humanized anti-IL5 mAb that neutralize IL-5. Both biologicals are already approved by the European and US Food and Drug Association (FDA) for its use in the treatment of severe eosinophilic asthma.

A phase II trial showed that reslizumab, at a dose of one single intravenous injection of 3 mg/kg, significantly reduces blood eosinophil counts and nasal IL-5 levels in patients with NP [88]. Individual NPs score improved for up to 4 weeks in only 50% of the patients. Additional post hoc analysis could identify a subpopulation of responders characterized by increased IL-5 levels in nasal secretions (i.e. >40 pg/mL).

A phase II trial with mepolizumab showed, similar to reslizumab, a reduction of blood eosinophil count paralleled by decreased levels of IL-5 in serum and nasal secretions of patients with CRSwNP [89]. However, nasal IL-5 and nasal total IgE were not significantly altered. More important, a reduction of the NP score was seen in patients with severe and/or recurrent NP after treatment with two single intravenous injections of 750 mg of Mepolizumab with an interval of 4 weeks. This study could however not support the association between responders and increased IL-5 levels, which presumably is due to a small sample size. A sustained effect for up to 36 weeks after treatment with mepolizumab was seen in the responder group, suggesting its long-term effect.

Reslizumab and Mepolizumab are both safe and well-tolerated in patients with CRSwNP. After treatment cessation rebound eosinophilia was reported, but this phenomenon seemed to occur without major exacerbation symptoms [88]. Studies with larger sample size, long treatment duration and follow-up are needed to determine the optimal treatment scheme for clinical use [89].

Lastly, it is worth noting that benralizumab is a humanized mAb against the highly expressed IL-5Rα receptor on eosinophils [90]. Its efficacy and safety in uncontrolled asthma with eosinophilia has been demonstrated in a phase III trial. So far, no studies are published on its use in upper airway diseases.

#### Targeting IL-4/IL-13 -pathway

IL-4 and IL-13 can be seen as sibling cytokines as they share the IL-4Rα subunit to form a fully functional IL-4 (with common γC subunit) or IL-4 and IL-13 (with IL-13Rα subunit) receptor [91]. This explains their mutual and important role in the type 2 inflammation.

Dupilumab, a fully human anti-IL4Rα mAb, is designed to interfere with this pathway. It has been proven to be effective in the treatment of atopic dermatitis and asthma, in which it also improved sinonasal symptoms [92, 93]. Recently a phase II trial evaluating dupilumab in the treatment of uncontrolled CRSwNP was published by Bachert et al. [94]. Adding dupilumab subcutaneously once every week to intranasal corticosteroid treatment showed improved endoscopic NP score, CT score (Lund–Mackay scoring system), QoL and major symptoms such as loss of smell, nasal obstruction or congestion and nocturnal awakenings. This effect remained up to 16 weeks after treatment cessation. Further studies are needed to assess longer treatment duration and direct comparison with other type 2 biological treatments.

#### Other type 2 directed therapies

Chemoattractant receptor homologous molecule on Th2 cells CRTH2 is responsible for eosinophil, basophil and lymphocyte recruitment upon PGD2 release of activated mast cells. The CRTH2-antagonist, OC000459, showed improvement of nasal and ocular symptoms in grass pollen allergic patients after grass pollen provocation [95]. Another CRTH2 antagonist, BI 671800, reduced total nasal symptoms in patients with seasonal allergic rhinitis after grass pollen provocation [96]. A comparative study including 146 patients found that its efficacy on nasal symptoms score was superior to montelukast, but inferior to intranasal fluticasone furoate [96].

GATA-3 is an important transcription factor and considered to be a ‘master switch’ of type 2 inflammation. It is responsible for the differentiation of Th0 cells towards Th2 cells and promotes the production of IL-4, IL-5 and IL-9 [97]. Significantly higher levels of GATA-3 mRNA has been documented in eosinophilic NP [98]. The use of a GATA-3 specific DNAzyme in the treatment of allergic asthma has been investigated in a phase IIb study, which reported a significant attenuation of asthmatic responses after allergen provocation, together with an attenuation of Th2-regulated inflammation [99]. So far, these targeted treatments have not been investigated in rhinitis or in CRS.

Siglec-8 is a cell surface receptor selectively expressed on mast cells, eosinophils and basophils [100]. Currently, a phase II study is ongoing to evaluate the efficacy of AK001, a monoclonal antibody targeting siglec-8, in patients with CRSwNP.

#### Allergen immunotherapy (AIT)

AIT is the only potential curative treatment option for IgE-mediated allergic diseases. The mechanisms of action of AIT include very early desensitization of mast cells and basophils, early generation of allergen-specific regulatory T cells (Treg) and B cells (Breg), suppression of Th2 and Th1 cells and late decrease of IgE/IgG4 ratio, tissue mast cell count and eosinophil counts [101]. This results in a reduced type 1 hypersensitivity reaction upon specific allergen exposure.

Currently there are two types of AIT applied in clinical practice, i.e. subcutaneous and sublingual immunotherapy AIT (respectively SCIT and SLIT). Despite the fact that both types of AIT have proven efficacy for different types of allergens, it is difficult to predict response to this treatment. Nonetheless, different biomarkers have been evaluated to identify AIT-responsive endotypes.

Several studies have shown that increased IgG4 levels in serum are associated with clinical improvement [102, 103]. Determination of allergen-specific IgE and IgG4 antibody levels via microarrays has been proposed as a potential biomarker to monitor AIT. Wollman et al. demonstrated that an IgG4 induced reduction of allergen-specific IgE binding has a high predictive value of 90% for clinical improvement. This assumption is based on the correlation between decreased allergen-specific IgE binding on Immuno Solid-phase Allergen Chip (ISAC) microarray and increased nasal tolerance in provocation tests [104]. Another clinical study performed by Schmid et al. showed that pre-treatment-specific IgE levels on ISAC microarray could predict the induction and magnitude of the IgG4 response during SCIT. Moreover, elevated IgE levels before treatment is thought to be a prerequisite for induction of IgG4 blocking antibodies. In addition IgE and IgG4 levels correlate with other functional immunological changes such as facilitated antigen binding, basophil sensitivity and clinical symptoms score [105].

Therefore, measuring IgE levels on serum samples before and after updosing could be used as a biomarker for early monitoring of AIT. This could be particularly useful in patients with uncertain clinical improvement upon AIT. For example, when IgE levels are decreased, AIT can be pursued aiming at further reduction of IgE. On the other hand, when no change or increase of the IgE levels or absence of IgG4 blocking antibodies is observed AIT should be modified or discontinued.

Another biomarker was proposed by Shamji et al. who demonstrated that ex vivo basophil hyporesponsiveness to allergen, confirmed with flow cytometry for intracellularly labelled diamine oxidase on blood samples, is useful to monitor efficacy and induction of allergen-specific tolerance during AIT [106]. Suppression of basophil responsiveness and subsequent histamine release was correlated with lower AR symptoms scores.

Several attempts are made to establish biomarkers for monitoring AIT. It appears essential that the proposed biomarkers should be compared with each other in regards to their predictive value as well as their reproducibility, complexity of measurement and cost-effectiveness. Further research towards biomarkers are needed and will generate essential information to create novel and improved AIT models.

### Therapies targeting non-type 2 inflammation

Development of novel therapies targeting non-type 2 inflammation seems more challenging compared with type 2 inflammation. Different biologicals have been investigated but these showed only little or no improvement in clinically relevant asthma outcome parameters.

CXC chemokine 2 receptor (CXCR2) antagonists (e.g. AZD5069, SCH527123) target the CXCR2 receptors on neutrophils and prevent their activation through the chemokine IL-8. Their efficacy has been investigated in the treatment of severe asthma, in which no clinical improvement was seen, despite reduction of neutrophils in sputum and blood [107, 108].

Brodalumab is a human anti-IL17A mAb designed to target IL-17A, a cytokine that is associated with neutrophilic inflammation and corticosteroid resistance. A trial of brodalumab in patients with uncontrolled moderate to severe asthma, without being selected for neutrophilic inflammation, reported no improvement of symptoms or lung function [109].

So far, no trials evaluating targeted treatments of non-type 2 inflammation have been conducted in rhinitis or CRS. The relative poor evolution in the development of biologicals targeting non-type 2 inflammation indicates that the non-type 2 inflammation needs further research to identify distinct subendotypes.

### Therapies targeting neurogenic activation

#### Targeting TRPV1 pathway

Capsaicin, the pungent substance in red pepper responsible for a burning sensation, has been proven to be effective in reducing NHR symptoms in IR [57, 110]. Capsaicin activates the TRPV1 channel which leads to influx of Ca^2+^ resulting in neuronal excitation and release of neuropeptides, followed by a long-lasting refractory period, during which the neurons are not responsive anymore to a broad range of stimuli. Its therapeutic effect is thought to be due to the above-mentioned de-functionalization and/or degeneration of C-fibers by massive Ca^2+^ influx [57]. Significant and long-term reduction (up to 9 months after treatment cessation) of NHR symptoms have been reported [111].

To optimize the treatment with capsaicin a study was performed comparing two regimens: application of five doses of capsaicin on one single day versus one dose of capsaicin every 2 days for 2 weeks. Both regimens seemed to be equally effective, but the former scheme is preferred as it is thought to enhance patient compliance [110].

An alternative therapeutic option is targeting the TRPV1 ion-channel with the selective TRPV1 antagonist SB-705498. Reduction of NHR symptoms due to intranasal application of SB-705498 has been documented [112] So far, no head-to-head trial has been performed comparing capsaicin and SB-705498.

### Therapies targeting epithelial barrier dysfunction

Restoring epithelial barrier function might reduce excessive penetration of allergens and/or harmful particles into the submucosal space, ultimately impeding continuous activation of the immune system and subsequent symptoms. Interfering with Toll like receptor or epidermal growth factor receptor signaling has been demonstrated to have barrier modulating capacity in different in vitro and murine studies (reviewed in Steelant et al.) [113]. Whether these treatments might also lead to clinical improvement of symptoms needs to be investigated.

Recent findings suggest that the therapeutic effect of locally applied corticosteroids might also result from their ability to enhance barrier integrity [62]. Since decades, corticosteroids are part of the standard treatment of the upper and lower airway diseases because of their anti-inflammatory properties [114, 115]. It was recently demonstrated that corticosteroids upregulate tight junction expression and thereby restore barrier function both in primary epithelial cell cultures and in a model of house dust mite (HDM)-induced allergic airway inflammation [62]. In addition, HDM AR patients taking inhaled steroids showed reduced epithelial permeability compared to steroid-naive patients [62].

## Current challenges in endotype-driven treatments

The recently gained insight into different pathophysiological mechanisms of rhinitis and CRS has been the driving force for the development of targeted therapies for patients with chronic upper airway disease. This evolution instigates the development of strategies to identify those patients who are most likely to benefit from these therapies. This so called endotype-driven treatment is one of the 4 pillars of precision medicine. Implementation of the principles of precision medicine into the management of airway diseases is a major challenge for the next decade [116].

Disease heterogeneity needs to be further explored and will lead to the discovery of new biomarkers that serve as a unique signature for a particular endotype. So far, important progresses have been made in identifying endotypes, of which type 2 inflammation is well ahead of other endotypes. Non-type 2 inflammation, the neurogenic endotype and barrier dysfunction needs further untangling and exploration.

So far, only a limited number of biomarkers are ready for use in clinical practice, such as blood eosinophils or serum specific and total IgE. Biomarkers should be pathway-specific, easy to measure, reproducible and affordable. The analysis of mediators in nasal secretions for endotyping is promising but requires further investigation. The biomarkers discussed in this article are mostly used in a research setting and represent potential biomarkers that need to be qualified and validated. An ideal strategy to reach biomarker identification, qualification and validation is through large-scale multi-center studies implying cooperation and standardization of laboratories and databanks all over the world. That way all efforts made towards precision medicine will be recognized more efficiently for implementation.

Besides ameliorating treatment approaches for the individual patients, precision medicine is thought to help in resolving the socio-economic burden of upper airway diseases [116]. It is believed that the reduction of the socio-economic burden due to endotype-driven treatment will outweigh the investments made in research of endotypes and development of novel treatment agents. Whilst the economic aspect is a motive for development of endotype-driven treatments, it is the main challenge at the same time. Considerable investments are required to conduct research on endotypes, biomarkers and biologicals. Raising political awareness about the allergy epidemics and its socio-economics costs is desired and will facilitate implementation in clinical practice. Because of the high cost associated with biological treatment, these molecules will in first place be retained for patients with severe and uncontrolled disease.

Three crucial prerequisites are identified to drive the next step in endotype-driven therapy:

- the demonstration of the predictive value of biomarkers in rhinitis and CRS, guiding the clinician in applying precision medicine in practise
- the recognition and better understanding of the contribution of one or more immunologic pathways or endotypes to the disease phenotype
- the demonstration of targeted treatment having superiority in clinically relevant outcomes and inferiority in cost over existing treatment options.

## Conclusion

This review provides an overview of the efforts made towards endotype-driven treatments in upper airway diseases. Current knowledge about type 2 inflammation is well ahead of other endotypes. Type 2 targeted treatments with monoclonal antibodies against IgE, IL5 and IL4Rα have been proven to be effective in chronic upper airway diseases supporting the importance of this endotype. Neurogenic inflammation as causative mechanism for nasal hyperreactivity is also well established and treatment with capsaicin is proven to be effective in IR with hyperreactivity. Barrier dysfunction and non-type 2 inflammation still need to be investigated more extensively to support its importance in upper airway diseases. Endotype-driven treatment still needs to face multiple challenges before its implementation in daily practice.
